# Duplication of Peno-Scroto-Testicular Unit- A Rare Form of Caudal Duplication Syndrome

**Published:** 2013-10-11

**Authors:** Anjan Kumar Dhua, Shandip Sinha, Simmi Ratan, Satish Aggarwal

**Affiliations:** Department of Pediatric Surgery, Maulana Azad Medical College, New Delhi

**Dear Sir,**

Duplication of penis has been reported to occur once in every 5 to 6 million live births.[1] It presents as duplication of only glans to complete duplication of the phallus and usually associated with other anomalies like bifid scrotum, double bladder, vertebral anomalies and ano-rectal malformations etc.[2] With this backdrop, herein we present a newborn with complete duplication of the entire peno-scroto-testicular unit (PSTU) with renal anomalies.

A 31-weeks preterm male baby was delivered by cesarean section which was done due to fetal distress at another hospital. He was a product of non-consanguineous marriage and was second in birth order. Antenatal ultrasound revealed severe oligohydramnios and absent right kidney at 28 weeks of gestation. Postnatally, the patient required resuscitation and later developed severe respiratory distress. The patient had prominent infra-orbital folds and characteristics of Potter’s sequence. Patient was lethargic with cold periphery. Baby was placed under infant warmer and resuscitated; subsequently inotropic support was started. Respiratory distress was significant requiring ventilator support. Local examination of the genitalia showed complete duplication of the PSTU of comparable size, one placed over another, the lower one appeared to be rotated 900 with respect to the upper one, each harboring appropriate sized testes (Fig. 1). The unit that was relatively at normal site had marked peno-scrotal transposition but with normal sized testis. Urethra was catheterizable. The phallus of the lower unit appeared normal but had an atretic meatus and could not be negotiated. Posteriorly, a patulous anus with normal caliber was found. Blood investigations revealed metabolic acidosis and hyperkalemia. Blood urea was 65 mg% and serum creatinine 2 mg%. Ultrasonography revealed agenesis of right kidney; left kidney was replaced by a multicystic structure. Bladder was not discernible. Echocardiography done showed a large muscular VSD. Sonogram of the spine revealed diplomyelia. A VCUG could not be done due to critical condition of the patient. Surgery was not offered as to expected fatal outcome. The baby expired after 24 hours due to respiratory failure. Parents refused autopsy.

**Figure:**
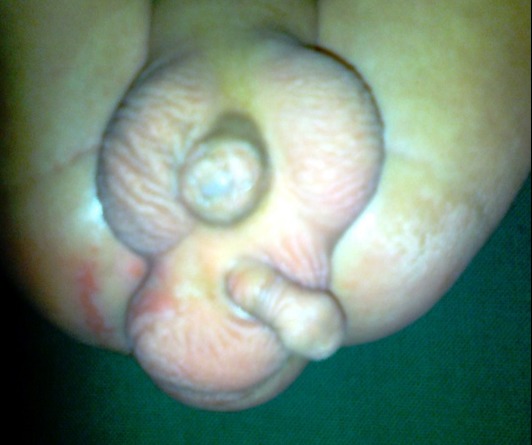
Figure 1: Complete duplication of peno-scroto-testicular units with penoscrotal transposition in the upper moiety and two testis oriented in a sagittal plane in the lower moiety. Also note perianal excoriation due to a posteriorly pushed and lax anal sphincter (hidden by the lower moiety).

Diphallia is a rare anomaly. Wecker first described it in 1609.[3] Rarely does diphallia occur in isolation. Dominguez et al coined the term caudal duplication syndrome (CDS) that resulted from an insult to the caudal cell mass and hindgut during 4th week of gestation. It encompasses complex malformations of spine, external genitalia, and lower urinary and reproductive structures.[4] In our case there was duplication of PSTU, diplomyelia, and renal anomalies. Urinary bladder and colonic duplication could not be ruled out as the baby expired within a few hours of presentation. This case may be considered a rare form of CDS with external manifestation as PSTU duplication. The additional involvement of testis in the spectrum, however, is worth noting. Renal anomalies in association with diphallus are rare. Mirshemirani et al reported complete diphallus, exstrophy of bladder, and unilateral renal agenesis. [5] Kardasevic et al reported a neonate with diphallus, duplicated bladder, and duplicated right ureter along with the right pelvicalyceal system.[6] With regards to scrotal anomalies, accessory scrotum has been described in literature, but they are usually empty and do not contain testicular tissue.[7] In our patient the renal anomalies were incompatible with life. Treatment is individualized and consists of surgical removal of the aberrant phallus after careful evaluation of the anatomic relationships of the various related structures.

## Footnotes

**Source of Support:** Nil

**Conflict of Interest:** None declared

